# Convergent Validity and Exploratory Discrimination of Two-Dimensional Facial Photographic Measures for Clinician-Rated Facial Asymmetry in Young Mongolian Adults

**DOI:** 10.7759/cureus.116961

**Published:** 2026-09-26

**Authors:** Bolormaa Sainbayar, Tsend-Ayush Batbayar, Nursabi Khizatkhan, Dariimaa Ganbat

**Affiliations:** 1 Department of Orthodontics, School of Dentistry, Mongolian National University of Medical Sciences, Ulaanbaatar, MNG; 2 Graduate School of Medical Science, Mongolian National University of Medical Sciences, Ulaanbaatar, MNG

**Keywords:** chin deviation, convergent validity, facial asymmetry, mongolian adults, orthodontics, photogrammetry, receiver operating characteristic, visual analog scale

## Abstract

Background: Frontal facial photography is accessible, non-ionizing, and routinely available in orthodontic practice, but the extent to which simple two-dimensional measures correspond to clinician-rated facial-asymmetry severity remains incompletely characterized.

Materials and methods: This cross-sectional secondary analysis included 77 Mongolian adults aged 18-35 years. Five specialist orthodontists assessed frontal photographs using a 100-mm visual analog scale (VAS); the archived dataset retained three ordinal severity categories and five per-rater category ratings. Inter-rater agreement was quantified with Fleiss kappa. Eleven objective soft-tissue measures were evaluated using Spearman rank correlation with 95% bootstrap confidence intervals (CIs) and Benjamini-Hochberg false-discovery-rate (FDR) adjustment. Exploratory receiver operating characteristic (ROC) analysis assessed FDR-significant measures for distinguishing moderate-to-severe from normal/minimal asymmetry.

Results: The sample included 21 men and 56 women (mean age 23.01±5.28 years); 39 participants had normal/minimal, 22 moderate, and 16 severe asymmetry. Exact-category inter-rater agreement was modest (Fleiss kappa=0.360, 95% CI 0.258-0.453). Chin deviation showed the strongest association with increasing severity (rho=0.492, 95% CI 0.286-0.668; q<0.001), followed by nasal deviation (rho=0.378; q=0.004), lip cant (rho=0.293; q=0.036), and ear-level cant (rho=0.280; q=0.037). Chin deviation had the highest exploratory area under the ROC curve (AUC) (0.709, 95% CI 0.606-0.803); a threshold of ≥1.5° yielded 44.7% sensitivity and 94.9% specificity. The age- and sex-adjusted ordinal odds ratio (OR) for chin deviation was 2.67 per 1° increase (95% CI 1.64-4.34), and, in a post hoc severe-versus-normal/minimal analysis (16 severe and 39 normal/minimal participants), the AUC was 0.811. All cut-offs and subgroup estimates are preliminary, hypothesis-generating results requiring prospective external validation.

Conclusions: Selected two-dimensional measures, particularly chin deviation, showed modest-to-moderate convergence with clinician-rated asymmetry. High specificity but limited sensitivity supports their use as quantitative adjuncts rather than stand-alone screening tests. These results reflect convergence with clinician perception, not criterion validity against three-dimensional anatomy; external validation and study-specific measurement-reliability data are needed before any of the internally selected operating points are interpreted as clinical thresholds.

## Introduction

Facial asymmetry is common and may involve the skeletal framework, dentition, soft tissues, or a combination of these components. Mild asymmetry is often visually acceptable, whereas more pronounced asymmetry can influence orthodontic diagnosis, treatment planning, and facial esthetics [1-3]. Visible soft-tissue asymmetry does not necessarily mirror underlying skeletal asymmetry because soft-tissue thickness and contour may partially compensate for, or accentuate, hard-tissue differences [1,3].

Clinical perception is threshold-dependent. Studies using manipulated two-dimensional and three-dimensional facial images have shown that orthodontists, other dental professionals, and lay observers differ in the magnitude and location of asymmetry they detect, with chin position and lip cant among the features that strongly influence perceived asymmetry [4-8]. Clinician perception is therefore a clinically relevant comparator, but it is not a definitive anatomical gold standard.

Frontal photography remains valuable because it is inexpensive, non-ionizing, repeatable, and already part of routine orthodontic records. Landmark-based photogrammetry can quantify facial relationships when acquisition and head positioning are controlled [9]. Photographic angular and proportional measurements have previously been demonstrated to capture significant soft-tissue variations associated with subjective facial assessments [10]. However, two-dimensional images compress three-dimensional anatomy into a single plane and remain sensitive to head posture, camera geometry, and landmark identification [11,12]. Three-dimensional surface and cone-beam computed tomographic approaches can quantify regional asymmetry more comprehensively, but they require additional equipment, expertise, and, for cone-beam computed tomography, ionizing radiation [13-17]. A carefully characterized two-dimensional method therefore remains clinically relevant as a low-burden adjunct when its limitations are explicit.

The primary objective of this secondary analysis was to evaluate the convergent validity of predefined two-dimensional facial photographic measures against subjective clinician-rated facial-asymmetry severity, a clinically relevant perceptual comparator rather than an anatomical gold standard. The secondary objective was to explore whether measures supported by the ordinal analysis could discriminate normal/minimal from moderate-to-severe perceived asymmetry. We hypothesized that lower face and midline deviation measures would show the strongest correspondence with specialist ratings.

Preliminary group-level soft-tissue findings from this dataset were presented at the 63rd Annual Scientific Conference of the School of Dentistry, Mongolian National University of Medical Sciences (MNUMS), Ulaanbaatar, Mongolia, on April 12, 2021. A related Mongolian-language article based on the source thesis and the same underlying participant cohort was subsequently published in a local Mongolian journal without a digital object identifier (DOI) [18]. The present article is a distinct secondary analysis: the participant-level rank-correlation, multiplicity-adjusted inference, inter-rater agreement, receiver operating characteristic (ROC), age/sex-adjusted ordinal regression, and sensitivity analyses reported here were not part of the prior publication or thesis presentation, and no tables, figures, or primary analytical results from the previous article are reproduced here.

## Materials and methods

Study design, setting, and ethics

This cross-sectional secondary analysis used participant-level data collected for a master's thesis within the parent project titled “Distribution, Clinical Characteristics, Interrelationships, and Treatment of Maxillofacial Disorders and Sleep Disorders in the Mongolian Population” (unpublished data: Tsolmon J. Distribution, Clinical Characteristics, Interrelationships, and Treatment of Maxillofacial Disorders and Sleep Disorders in the Mongolian Population. 2020-2025). The study was conducted through the Department of Orthodontics, School of Dentistry, MNUMS, Ulaanbaatar, Mongolia, and is reported in accordance with the Strengthening the Reporting of Observational Studies in Epidemiology (STROBE) recommendations [19]. The MNUMS Biomedical Ethics Review Committee approved the study (Protocol No. 2021/3-04; March 19, 2021), and a subsequent ethics review confirmed continued compliance (Protocol No. 2022/4-01; January 21, 2022). All participants provided written informed consent for study participation and research use of their clinical photographs. No identifiable facial photographs are reproduced in this article. 

Participants

The parent project included 2,000 individuals aged 13-65 years. The thesis-specific analysis included 77 adults who met its eligibility criteria and had frontal photographs suitable for facial-asymmetry assessment. Inclusion criteria were age 18 years or older, permanent dentition, and an acceptable frontal facial photograph. Exclusion criteria were cleft lip and/or palate, facial paralysis, temporomandibular joint pain or symptoms, and partial or complete edentulism. The resulting cohort ranged from 18 to 35 years and comprised Mongolian adults recruited through the single study center. Age and sex were the demographic variables retained in the archived analytic dataset; ethnicity, residence, education, and socioeconomic indicators were not separately recorded and therefore could not be reported. No additional participants were recruited, and there were no missing values for the archived final visual analog scale (VAS) category or the 11 photographic measures. No new a priori sample-size calculation was performed because this secondary analysis used the complete eligible thesis cohort; precision is therefore reported with confidence intervals rather than retrospective power calculations.

The same 77 participants also contributed to a companion analysis addressing a different research question, namely the association of sagittal and transverse malocclusion with clinician-rated facial-asymmetry severity (unpublished data: Sainbayar B, Tsend-Ayush B, Khizatkhan N, Ganbat D. Association of Sagittal and Transverse Malocclusion With Clinician-Rated Facial Asymmetry Severity in Young Mongolian Adults: A Cross-Sectional Study. 2026). The present article does not analyze malocclusion exposures, and no tables, figures, or primary malocclusion results from the companion analysis are reproduced here.

Frontal photographs and clinician-rated asymmetry

Archived thesis materials specified eligibility criteria for frontal photographs rather than a fully recoverable camera-acquisition protocol. Photographs were eligible when both ears were visible, right-left positioning relative to the lateral orbital region and hairline was comparable, and the lips and facial musculature were relaxed. The archived records did not preserve the camera body or lens, subject-to-camera distance, lighting configuration, or a formal natural-head-position procedure; these technical details were therefore not reconstructed.

Five specialist orthodontists assessed the 77 photographs using a 100-mm VAS [20] on two occasions separated by two weeks. Five relatively symmetric and five markedly asymmetric photographs were used as visual anchors, following a published photogrammetric VAS approach [20]. The thesis defined three regions: normal/minimal asymmetry (0-33.3), moderate asymmetry (33.4-66.7), and severe asymmetry (66.8-100). The archived secondary-analysis workbook retained one ordinal category from each of the five orthodontists and one final participant-level category, but not the complete continuous 0-100 scores from both sessions. Continuous-score correlations and test-retest ICCs therefore could not be reconstructed.

For 75 of 77 participants, the five archived rater categories had a unique majority of at least three raters, and the archived final category matched that majority in all 75. Two participants had 2-2-1 ties across the three categories; both had been stored as severe in the archived final variable. The original tie-adjudication rule was not preserved, so the archived final categories were retained without post hoc reclassification. Exact-category inter-rater agreement among the five archived ratings was quantified with Fleiss kappa.

Photographic measurements

Following the Farkas anthropometric approach [21], the thesis used 11 soft-tissue landmarks and 10 reference lines to derive nine angular measures and two gonion-based asymmetry indices. Eye-level, ear-level, lip, and mandibular-angle cants were defined as the angles between the horizontal reference and the corresponding bilateral landmark lines. Nasal deviation was the angle between the facial vertical midline and the nasion-pronasale line; chin deviation was the angle between the vertical midline and the O-point-menton line, where the O-point represented the intersection of the facial vertical and horizontal reference lines. Ramus inclination difference and mandibular body inclination difference represented right-left differences in the inclinations of the preauricular-gonion and gonion-menton lines, respectively, and mandibular angle difference represented the right-left difference in preauricular-gonion-menton angles. Horizontal and vertical gonion asymmetry indices expressed proportional right-left differences as $\frac{\left|R-L\right|}{\operatorname{mean} (R,L)}\times 100$.

The detailed operational definitions are provided in the Appendices, and the measurement geometry is illustrated schematically in Figure 1.

**Figure 1:**
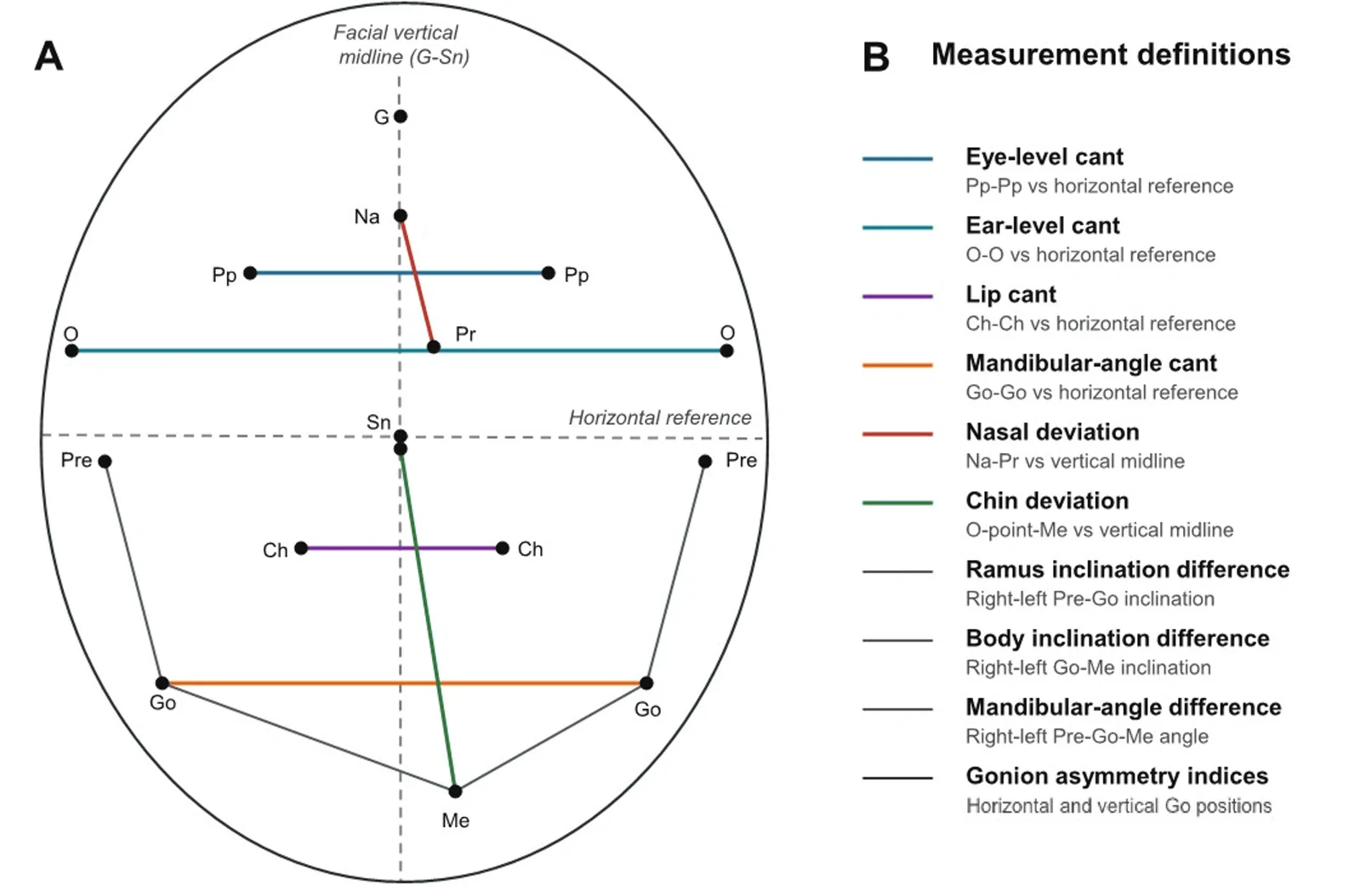
Schematic measurement geometry for the 11 objective photographic measures. The facial vertical midline was defined by G-Sn, and the horizontal reference was drawn perpendicular to it through the O-point. Bilateral cant was the acute angle between the horizontal reference and the line joining the paired landmarks. Nasal and chin deviations were defined as the acute angles of Na-Pr and O-point-Me, respectively, relative to the facial vertical midline. Right-left ramus, body, and mandibular-angle differences were calculated from paired Pre-Go, Go-Me, and Pre-Go-Me geometry; Go indices used proportional right-left distances. G, glabella; Sn, subnasale; Na, nasion; Pr, pronasale; Pp, pupil center; O, otobasion/inferior ear-lobe attachment point; Ch, cheilion point; Me, menton; Go, gonion point; Pre, preauricular point; O-point, intersection of the facial vertical and horizontal reference lines. Credits: Figure created by the authors in Microsoft PowerPoint 2019 (Microsoft Corporation, Redmond, USA). The image is an explanatory schematic and does not represent a participant photograph.

The source thesis stated that one investigator manually performed all 11 photographic measurements twice. The archived workbook available for the present analysis retained only the final participant-level study value for each measure, not the paired replicate measurements; consequently, study-specific intra-examiner intraclass correlation coefficients (ICCs), standard errors of measurement, and Bland-Altman limits of agreement could not be recalculated.

For replication of the archived definitions, landmarks were operationalized in a fixed sequence: (i) identify glabella (G) and subnasale (Sn) and draw the facial vertical midline; (ii) draw the perpendicular horizontal reference through their intersection point (O-point); (iii) mark bilateral pupil centers (Pp), otobasion/inferior ear-lobe attachment points (O), cheilion points (Ch), soft-tissue gonion points (Go), and preauricular points (Pre), together with nasion (Na), pronasale (Pr), and menton (Me); (iv) construct the bilateral Pp-Pp, O-O, Ch-Ch, and Go-Go lines and record their acute angles to the horizontal reference; (v) construct Na-Pr and O-point-Me and record their acute angles to the vertical reference; (vi) construct bilateral Pre-Go and Go-Me segments and the Pre-Go-Me angles, then calculate absolute right-left differences; and (vii) calculate horizontal and vertical gonion asymmetry as $\frac{\left|R-L\right|}{\operatorname{mean} (R,L)}\times 100$.

This reconstruction improves procedural transparency but does not recover the unavailable acquisition settings or replicate measurements.

Statistical analysis

Continuous photographic measures were summarized by median and interquartile range (IQR) because several distributions were zero-inflated or non-normal. Participant age was summarized overall as mean ± standard deviation (SD) and by severity group as median (IQR). Age across the three VAS categories was compared with the Kruskal-Wallis test, and sex distribution was compared with an exact 3×2 test.

The primary validity analysis treated the final VAS category as an ordinal severity variable coded one to three and calculated Spearman rank correlations (rho) between severity and each of the 11 photographic measures. Ninety-five percent confidence intervals (CIs) for rho were estimated using 5,000 nonparametric participant-level bootstrap resamples with random seed 20260906. Raw two-sided P values were adjusted across the 11 primary correlations using the Benjamini-Hochberg false-discovery-rate (FDR) procedure; q<0.05 was considered statistically significant. Because only three ordered severity levels were retained, ties were frequent and the analysis contained less information than a continuous-score analysis; this limitation was addressed through effect estimates, CIs, and sensitivity analyses rather than by reconstructing unavailable VAS values.

For the secondary exploratory discrimination analysis, moderate and severe categories were combined (n=38) and compared with normal/minimal asymmetry (n=39). ROC analyses were restricted to measures retaining FDR-adjusted significance in the primary ordinal analysis. AUCs and 95% CIs were estimated using 5,000 stratified bootstrap resamples. Study-specific cut-offs were selected with the Youden index. Sensitivity and specificity are reported with Wilson 95% CIs. For the chin-deviation threshold, positive predictive value (PPV), negative predictive value (NPV), and positive and negative likelihood ratios were calculated relative to the observed 49.4% prevalence of moderate-to-severe clinician-rated asymmetry.

Four post hoc robustness analyses were performed and interpreted as exploratory without multiplicity adjustment: sex-stratified Spearman correlations for the four FDR-significant measures; ROC analysis comparing severe asymmetry (n=16) directly with normal/minimal asymmetry (n=39), excluding the moderate group; separate proportional-odds cumulative-logit models for each FDR-significant measure adjusted for age and sex; and a tie-exclusion analysis omitting the two participants whose five archived rater categories formed 2-2-1 ties. These analyses were used to assess robustness rather than to develop a prediction tool.

Inter-rater agreement was quantified with Fleiss kappa and a 95% CI from 5,000 participant-level bootstrap resamples. Original thesis analyses were performed with IBM SPSS Statistics for Windows, Version 21.0 (IBM Corporation, Armonk, USA). The present secondary analyses were performed in Python 3.13.5 (Python Software Foundation, Fredericksburg, USA) using SciPy 1.17.0, scikit-learn 1.8.0, NumPy 2.3.5, and statsmodels 0.14.6.

## Results

Participant characteristics and rater agreement

The 77 participants included 21 men (27.3%) and 56 women (72.7%), with a mean age of 23.01±5.28 years. The final VAS classification comprised 39 participants (50.6%) with normal/minimal asymmetry, 22 (28.6%) with moderate asymmetry, and 16 (20.8%) with severe asymmetry. Age increased across severity groups (Kruskal-Wallis H=9.41, P=0.009), whereas the sex distribution did not differ significantly by exact testing (P=0.060) (Table 1).

**Table 1:** Participant characteristics by clinician-rated facial-asymmetry severity. Age P value is from the Kruskal-Wallis test; sex P value is from an exact 3×2 contingency table test. Overall mean age was 23.01±5.28 years. IQR, interquartile range.

Characteristic	Overall (N=77)	Normal/minimal (n=39)	Moderate (n=22)	Severe (n=16)	P value
Age, years, median (IQR)	21.0 (19.0-25.0)	19.0 (18.0-23.0)	24.0 (21.0-28.75)	23.5 (20.0-28.0)	0.009
Men, n (%)	21 (27.3)	13 (33.3)	2 (9.1)	6 (37.5)	0.06
Women, n (%)	56 (72.7)	26 (66.7)	20 (90.9)	10 (62.5)	

Across the five archived ordinal ratings, 21 participants (27.3%) had unanimous classification and 40 (51.9%) had agreement by at least four of five orthodontists. A unique majority of at least three raters was present for 75 participants (97.4%). Fleiss kappa for exact-category inter-rater agreement was 0.360 (95% bootstrap CI 0.258-0.453), indicating meaningful but imperfect observer variability in the clinical comparator.

Distribution of objective photographic measures

Several cant and midline-deviation variables were concentrated near zero, whereas mandibular difference and gonion asymmetry measures showed wider distributions (Table 2).

**Table 2:** Distribution of the 11 objective photographic measures. IQR, interquartile range.

Measure	Unit	Median (IQR)	Range
Eye-level cant	degrees	0.50 (0.00-1.50)	0-3.5
Ear-level cant	degrees	1.00 (0.00-1.50)	0-3.5
Lip cant	degrees	0.50 (0.00-1.00)	0-4.5
Mandibular angle cant	degrees	1.00 (0.50-2.00)	0-4.0
Nasal deviation	degrees	0.00 (0.00-0.00)	0-5.0
Chin deviation	degrees	0.00 (0.00-1.00)	0-7.0
Ramus inclination difference	degrees	2.00 (1.00-3.50)	0-8.0
Mandibular body inclination difference	degrees	1.50 (1.00-3.00)	0-8.0
Mandibular angle difference	degrees	2.00 (1.00-4.00)	0-10.0
Horizontal gonion asymmetry index	%	5.26 (2.66-8.57)	0-35.5
Vertical gonion asymmetry index	%	2.24 (0.03-4.87)	0-44.5

Convergent validity with ordinal clinician-rated severity

Four measures retained statistically significant positive monotonic associations with increasing clinician-rated severity after FDR adjustment (Table 3 and Figure 2). Chin deviation had the largest association (rho=0.492, 95% CI 0.286-0.668; q<0.001). Nasal deviation was next (rho=0.378, 95% CI 0.137-0.566; q=0.004), followed by lip cant (rho=0.293, 95% CI 0.070-0.503; q=0.036) and ear-level cant (rho=0.280, 95% CI 0.069-0.475; q=0.037). Eye-level cant had a nominal P=0.049 but did not remain significant after FDR adjustment (q=0.107). The remaining measures, including ramus inclination difference and both gonion asymmetry indices, showed no significant monotonic association.

**Table 3:** Spearman associations between objective measures and ordinal facial-asymmetry severity. FDR q values were calculated with the Benjamini-Hochberg procedure across all 11 primary correlations. CI, confidence interval; FDR, false discovery rate.

Measure	Spearman rho	95% bootstrap CI	Raw P	FDR q
Eye-level cant	0.225	0.001 to 0.446	0.049	0.107
Ear-level cant	0.280	0.069 to 0.475	0.013	0.037
Lip cant	0.293	0.070 to 0.503	0.010	0.036
Mandibular angle cant	0.188	-0.040 to 0.400	0.101	0.185
Nasal deviation	0.378	0.137 to 0.566	<0.001	0.004
Chin deviation	0.492	0.286 to 0.668	<0.001	<0.001
Ramus inclination difference	0.041	-0.190 to 0.266	0.720	0.881
Mandibular body inclination difference	0.107	-0.133 to 0.336	0.355	0.488
Mandibular angle difference	0.008	-0.214 to 0.236	0.946	0.989
Horizontal gonion asymmetry index	-0.002	-0.223 to 0.225	0.989	0.989
Vertical gonion asymmetry index	0.111	-0.106 to 0.324	0.338	0.488

**Figure 2:**
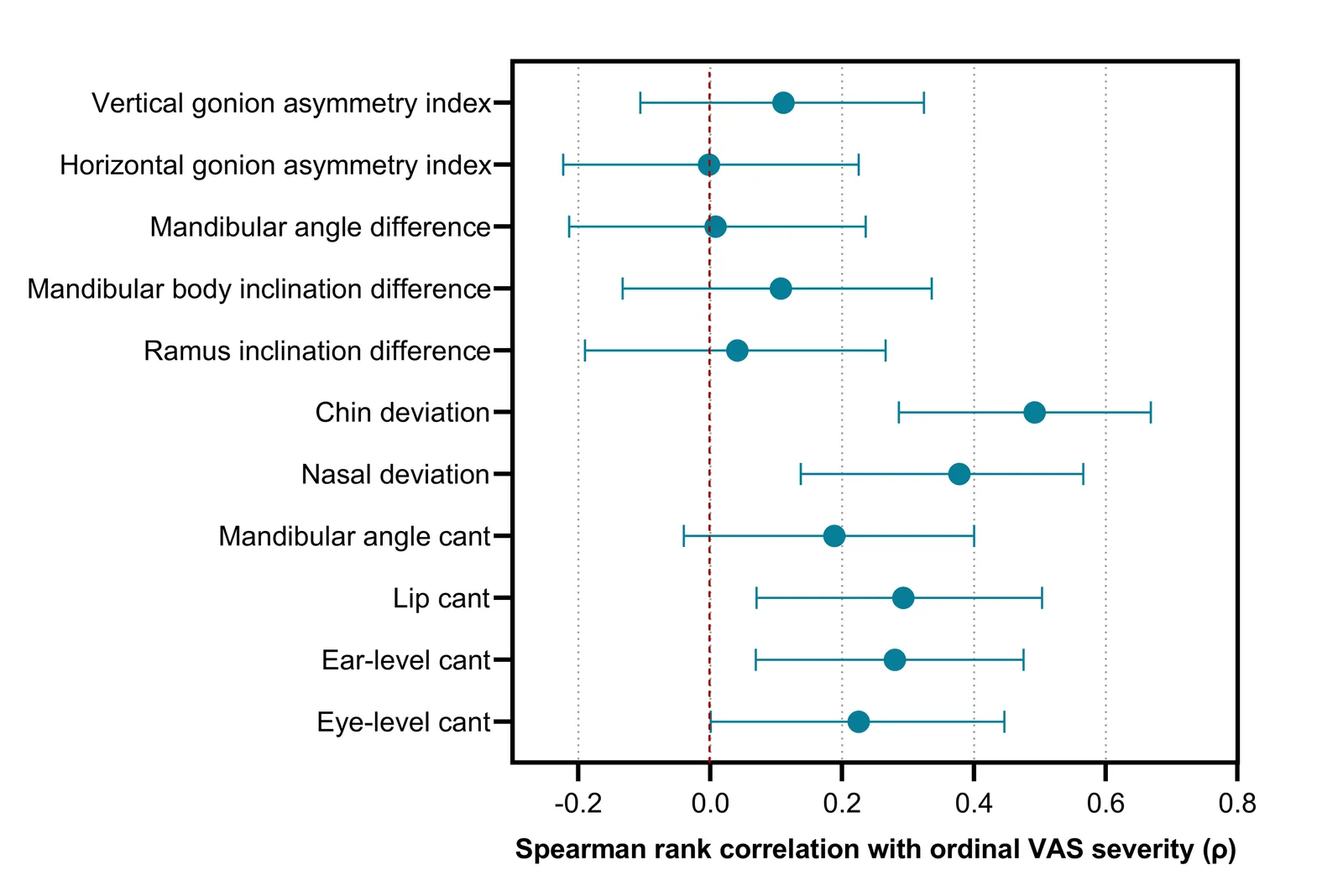
Association of objective photographic measures with clinician-rated facial-asymmetry severity. Points show Spearman rank-correlation coefficients (rho); error bars show 95% CIs from 5,000 participant-level bootstrap resamples. The dashed vertical line at rho=0 denotes no monotonic association. VAS, visual analog scale; CI, confidence interval.

Exploratory discriminative performance

ROC analysis was restricted to the four measures that retained FDR-adjusted significance in the primary ordinal analysis (Table 4 and Figure 3). Chin deviation had the highest area under the ROC curve (AUC) at 0.709 (95% CI 0.606-0.803). An exploratory threshold of ≥1.5° yielded sensitivity of 44.7% (95% CI 30.1%-60.3%) and specificity of 94.9% (95% CI 83.1%-98.6%). At the observed 49.4% prevalence of moderate-to-severe clinician-rated asymmetry, this threshold had a sample-specific PPV of 89.5% (95% CI 68.6%-97.1%), NPV of 63.8% (95% CI 50.9%-74.9%), positive likelihood ratio of 8.72, and negative likelihood ratio of 0.58. Ear-level cant had an AUC of 0.662, lip cant 0.637, and nasal deviation 0.598. The candidate thresholds generally favored specificity over sensitivity.

**Table 4:** Exploratory ROC performance for moderate-to-severe versus normal/minimal facial asymmetry. Cut-offs were selected by the Youden index in the same dataset and are exploratory; sensitivity and specificity CIs are Wilson intervals. AUC, area under the ROC curve; CI, confidence interval; ROC, receiver operating characteristic.

Measure	AUC	95% bootstrap CI	Exploratory cut-off	Sensitivity, % (95% CI)	Specificity, % (95% CI)
Chin deviation	0.709	0.606-0.803	≥1.5°	44.7 (30.1-60.3)	94.9 (83.1-98.6)
Ear-level cant	0.662	0.541-0.778	≥1.5°	50.0 (34.8-65.2)	79.5 (64.5-89.2)
Lip cant	0.637	0.517-0.752	≥1.5°	34.2 (21.2-50.1)	89.7 (76.4-95.9)
Nasal deviation	0.598	0.526-0.675	≥0.5°	23.7 (13.0-39.2)	94.9 (83.1-98.6)

**Figure 3:**
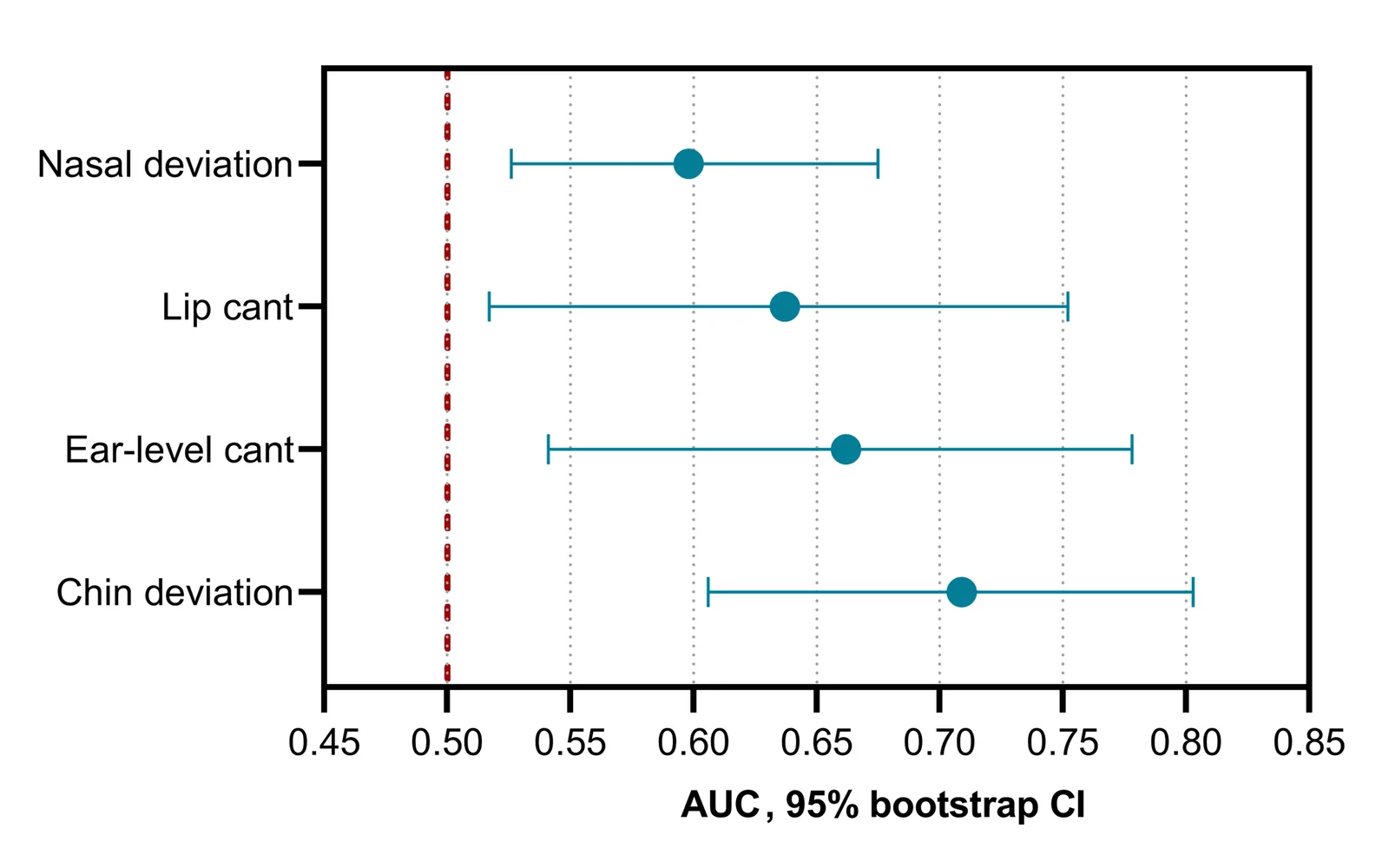
Exploratory discrimination of moderate-to-severe versus normal/minimal clinician-rated facial asymmetry. Points show AUC estimates; error bars show 95% CIs from 5,000 stratified bootstrap resamples. The dashed vertical line at AUC=0.50 denotes chance discrimination. AUC, area under the ROC curve; CI, confidence interval; ROC, receiver operating characteristic.

Robustness and sensitivity analyses

The main signal was not confined to the larger female subgroup or to the combined moderate/severe endpoint (Table 5). Chin deviation remained positively associated with severity in men (rho=0.442) and women (rho=0.516), although the male estimate was imprecise because only 21 men were included. In a post hoc severe-only comparison based on 16 severe and 39 normal/minimal participants and excluding the moderate group, chin deviation showed stronger but imprecisely estimated discrimination (AUC=0.811, 95% CI 0.668-0.929); lip cant, nasal deviation, and ear-level cant had AUCs of 0.716, 0.703, and 0.684, respectively. In exploratory proportional-odds models adjusted for age and sex, chin deviation, nasal deviation, and lip cant retained statistically significant associations with greater severity, whereas ear-level cant attenuated to borderline significance. Excluding the two 2-2-1 rater-tie cases did not materially alter the primary signal: chin deviation remained the strongest correlate (rho=0.455, P<0.001) and the moderate-to-severe versus normal/minimal AUC remained 0.694 (95% bootstrap CI 0.594-0.789).

**Table 5:** Robustness analyses for the four FDR-significant photographic measures. Sex-stratified correlations and adjusted models are exploratory and were not multiplicity-adjusted. The severe-only ROC analysis compares severe (n=16) with normal/minimal (n=39), excluding moderate cases. Ordinal ORs come from separate proportional-odds models adjusted for age and sex. AUC, area under the ROC curve; CI, confidence interval; FDR, false discovery rate; OR, odds ratio; ROC, receiver operating characteristic.

Measure	Men: rho (P)	Women: rho (P)	Severe vs normal/minimal AUC (95% CI)	Age/sex-adjusted ordinal OR per 1° (95% CI), P
Ear-level cant	0.506 (0.019)	0.192 (0.156)	0.684 (0.526-0.833)	1.57 (0.98-2.51), 0.058
Lip cant	0.227 (0.323)	0.308 (0.021)	0.716 (0.565-0.857)	2.14 (1.24-3.70), 0.007
Nasal deviation	0.467 (0.033)	0.351 (0.008)	0.703 (0.573-0.833)	6.35 (1.64-24.59), 0.007
Chin deviation	0.442 (0.045)	0.516 (<0.001)	0.811 (0.668-0.929)	2.67 (1.64-4.34), <0.001

## Discussion

The participant-level reanalysis identified a coherent but clinically modest pattern. Chin deviation showed the strongest monotonic correspondence with increasing clinician-rated severity and the best univariable discrimination of moderate-to-severe asymmetry. Nasal deviation, lip cant, and ear-level cant also remained significant after FDR adjustment. The severe-only analysis strengthened the chin-deviation signal, while sex-stratified results showed positive chin-deviation associations in both men and women. These findings support selected frontal photographic measures as quantitative adjuncts rather than stand-alone screening or diagnostic tests.

The prominence of chin deviation is clinically plausible. Observer-based studies consistently show that lower-face midline deviations can be conspicuous to both clinicians and lay observers, and lip cant also contributes to perception of facial imbalance [4-8,22]. This correspondence is consistent with evidence that objective facial measurements correlate with professional esthetic evaluations of facial balance [23]. More recent research confirms that the amount of chin deviation considered detectable or unacceptable depends on evaluator type and facial morphology, with orthodontists generally identifying smaller deviations than patients or lay observers [24]. Our internally selected 1.5° cut-off is therefore a preliminary, hypothesis-generating operating point rather than a universal perceptual or diagnostic threshold; it requires prospective external validation.

The threshold analysis adds practical nuance. Relative to the clinician-rated binary outcome, a chin deviation of at least 1.5° was highly specific but insensitive. Its sample-specific PPV was 89.5% and the positive likelihood ratio was 8.72, so a positive finding substantially increased the odds of a moderate-to-severe specialist rating in this sample. By contrast, the NPV was only 63.8% and the negative likelihood ratio was 0.58; absence of a ≥1.5° deviation therefore did not rule out clinically perceived asymmetry. This asymmetric performance supports confirmation rather than screening use.

Inter-rater agreement among the five archived ordinal ratings was modest (Fleiss kappa=0.360), although 97.4% of participants had a unique majority category. Observer variability is expected in perceptual assessment, and prior work has shown meaningful differences among professional and lay raters [4-8,22]. The present kappa estimate is important because it quantifies noise in the clinical comparator. It also reinforces the conceptual distinction between convergent validity against clinician perception and criterion validity against an objective anatomical standard.

The reanalysis also illustrates why group-comparison significance should not automatically be interpreted as convergent validity. The source thesis described between-group differences for several measures, including ramus inclination difference. In the participant-level ordinal analysis, however, ramus inclination difference showed virtually no monotonic association (rho=0.041; q=0.881). The present article therefore emphasizes ordered effect size, multiplicity-adjusted inference, and discriminative performance rather than reusing group-comparison P values as evidence of validity.

Age differed across the three VAS severity groups, whereas the sex distribution did not reach statistical significance. Exploratory adjustment for age and sex preserved the chin-, nasal-, and lip-deviation associations but attenuated ear-level cant. These analyses reduce concern that the principal findings were solely a consequence of the female predominance or age imbalance, but they remain post hoc and are not substitutes for a larger, balanced validation cohort.

Two-dimensional photogrammetry remains attractive because it is inexpensive, non-ionizing, and routinely available. A systematic review of facial-asymmetry imaging concluded that three-dimensional approaches offer more comprehensive and precise characterization, while two-dimensional methods remain useful in appropriate diagnostic contexts [25]. Three-dimensional assessments further demonstrate that facial soft-tissue asymmetry involves complex regional variation that planar projections cannot fully characterize [26]. More recent 3D surface studies also demonstrate high reliability when acquisition, registration, and operator procedures are standardized [13,16,17]. The current findings therefore support two-dimensional photographs as a low-burden adjunct, not as a replacement for three-dimensional surface imaging or cone-beam computed tomography when anatomical characterization is clinically indicated.

Strengths of this study include participant-level reanalysis of the original thesis cohort, use of five specialist orthodontists, transparent quantification of archived inter-rater agreement, 11 predefined photographic measures, FDR control across the primary correlations, bootstrap CIs, and prespecified restriction of the main ROC analysis to FDR-significant measures. The sensitivity analyses directly addressed the age/sex imbalance and the small severe group. Avoiding a multivariable prediction model also reduced overfitting risk in a modest dataset.

Several limitations remain. First, the sample was modest, single-center, and predominantly female, limiting generalizability. Second, the archived dataset retained three VAS severity categories rather than complete continuous 0-100 ratings; categorization reduces information and may reduce precision or attenuate observable associations. Third, although the thesis protocol states that objective photographic measurements were repeated twice by one investigator, paired replicate values were not retained in the archived workbook, so study-specific ICCs and Bland-Altman limits could not be calculated. Fourth, the archived materials did not preserve camera/lens specifications, subject-to-camera distance, lighting configuration, or a formal natural-head-position protocol, limiting technical reproducibility. Fifth, clinician-rated severity is an imperfect comparator rather than a three-dimensional anatomical gold standard. Sixth, ROC cut-offs were selected and evaluated in the same sample after candidate selection by ordinal association, so their performance is likely optimistic; the cut-offs are explicitly preliminary and hypothesis-generating and require prospective external validation before clinical use. Seventh, a related Mongolian-language article based on the same source thesis and underlying cohort was previously published in a local journal without a DOI [18]. Although the present article addresses a different research question and reports distinct participant-level analyses, the shared dataset creates potential concern for perceived overlap; this relationship is explicitly disclosed, and no tables, figures, or primary analytical results from the earlier publication are reproduced. Finally, the original rule for resolving two 2-2-1 rater ties was not recoverable; however, excluding those two cases did not materially change the primary correlation or ROC findings.

## Conclusions

In this sample of young Mongolian adults, chin deviation, nasal deviation, lip cant, and ear-level cant showed statistically significant monotonic correspondence with increasing clinician-rated facial-asymmetry severity after FDR adjustment. Chin deviation was the strongest individual correlate, remained robust after age/sex adjustment and tie-exclusion sensitivity analysis, and showed the best exploratory discrimination, particularly in the severe-only comparison.

At the internally selected, preliminary operating point, chin deviation was highly specific but insufficiently sensitive for screening. Standardized two-dimensional photogrammetry should therefore be considered a quantitative adjunct to clinical examination rather than a stand-alone diagnostic test. External validation in larger, demographically balanced cohorts, with recoverable measurement-reliability data and objective three-dimensional reference methods, is required before any clinical cut-off is adopted.

## Appendices

**Table 6:** Operational definitions of the 11 objective photographic measures G, glabella; Sn, subnasale; O-point, intersection of the facial vertical and horizontal reference lines; Pre, preauricular point. Credits: Table compiled by the authors using the archived thesis measurement protocol. The landmark nomenclature and anthropometric framework are based on Farkas [21].

Measure	Landmarks/reference line	Operational definition
Eye-level cant	Bilateral pupil centers (Pp); horizontal reference	Angle between the interpupillary line and the horizontal reference.
Ear-level cant	Bilateral otobasion/inferior ear-lobe attachment points (O); horizontal reference	Angle between the bilateral ear-level line and the horizontal reference.
Lip cant	Bilateral cheilion points (Ch); horizontal reference	Angle between the intercheilion line and the horizontal reference.
Mandibular angle cant	Bilateral soft-tissue gonion points (Go); horizontal reference	Angle between the bilateral gonion line and the horizontal reference.
Nasal deviation	Facial vertical midline (G-Sn); nasion (Na) to pronasale (Pr)	Angle between the facial vertical midline and the Na-Pr line.
Chin deviation	Facial vertical midline; O-point to menton (Me)	Angle between the facial vertical midline and the O-point-Me line.
Ramus inclination difference	Right and left Pre-Go lines; vertical midline	Absolute right-left difference between the inclinations of the two Pre-Go lines relative to the vertical midline.
Mandibular body inclination difference	Right and left Go-Me lines; vertical midline	Absolute right-left difference between the inclinations of the two Go-Me lines relative to the vertical midline.
Mandibular angle difference	Right and left Pre-Go-Me angles	Absolute right-left difference between the soft-tissue mandibular angles.
Horizontal gonion asymmetry index	Right and left gonion positions relative to the facial vertical midline	Proportional right-left difference in horizontal gonion position: $\frac{\left|R-L\right|}{\operatorname{mean} (R,L)}\times 100$.
Vertical gonion asymmetry index	Right and left gonion positions relative to the horizontal reference	Analogous proportional right-left difference in vertical gonion position: $\frac{\left|R-L\right|}{\operatorname{mean} (R,L)}\times 100$.
